# Deep Eutectic Solvents Comprising Organic Acids and Their Application in (Bio)Medicine

**DOI:** 10.3390/ijms24108492

**Published:** 2023-05-09

**Authors:** Tomasz Swebocki, Alexandre Barras, Amar Abderrahmani, Kamel Haddadi, Rabah Boukherroub

**Affiliations:** Univ. Lille, CNRS, Centrale Lille, Univ. Polytechnique Hauts-de-France, UMR 8520-IEMN-Institut d’Electronique de Microélectronique et de Nanotechnologie, 59000 Lille, France; alexandre.barras@univ-lille.fr (A.B.); amar.abderrahmani@univ-lille.fr (A.A.); kamel.haddadi@univ-lille.fr (K.H.)

**Keywords:** deep eutectic solvents, organic acids, drug delivery, enhancers, antimicrobial treatment

## Abstract

Over the last years, we observed a significant increase in the number of published studies that focus on the synthesis and characterization of deep eutectic solvents (DESs). These materials are of particular interest mainly due to their physical and chemical stability, low vapor pressure, ease of synthesis, and the possibility of tailoring their properties through dilution or change of the ratio of parent substances (PS). DESs, considered as one of the greenest families of solvents, are used in many fields, such as organic synthesis, (bio)catalysis, electrochemistry, and (bio)medicine. DESs applications have already been reported in various review articles. However, these reports mainly described these components’ basics and general properties without focusing on the particular, PS-wise, group of DESs. Many DESs investigated for potential (bio)medical applications comprise organic acids. However, due to the different aims of the reported studies, many of these substances have not yet been investigated thoroughly, which makes it challenging for the field to move forward. Herein, we propose distinguishing DESs comprising organic acids (OA-DESs) as a specific group derived from natural deep eutectic solvents (NADESs). This review aims to highlight and compare the applications of OA-DESs as antimicrobial agents and drug delivery enhancers—two essential fields in (bio)medical studies where DESs have already been implemented and proven their potential. From the survey of the literature data, it is evident that OA-DESs represent an excellent type of DESs for specific biomedical applications, owing to their negligible cytotoxicity, fulfilling the rules of green chemistry and being generally effective as drug delivery enhancers and antimicrobial agents. The main focus is on the most intriguing examples and (where possible) application-based comparison of particular groups of OA-DESs. This should highlight the importance of OA-DESs and give valuable clues on the direction the field can take.

## 1. Introduction

### 1.1. Brief Background and State-of-the-Art

**Figure 1:**
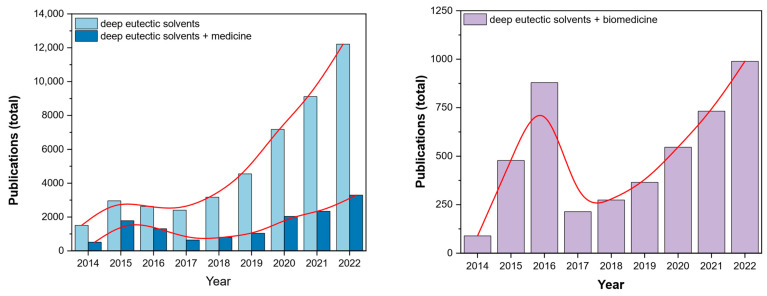
Number of papers published over the 2014–2022 period covering the interest in the field over the recent years (keywords: deep eutectic solvents, deep eutectic solvents + medicine, deep eutectic solvents + biomedicine, according to Digital Science & Research Solutions, Inc., London, UK).

Deep eutectic solvents (DESs) are a fascinating group of substances introduced to a broader scientific audience in 2003 by Abbott et al. [1]. Since then, DESs have brought more and more interest with observable ‘boom’ over recent years (Figure 1). Their medical-related applications contribute to 25–50% of the papers published in the last eight years. A similar trend was also observed for the number of works published in DESs in biomedicine, which mostly covers similar applications. Due to their negligible adverse effect on the environment, chemical composition and low cytotoxicity, they are considered a family of green solvents [2]. DESs have found their use in many fields of chemistry, such as catalysis [3], metal processing [4], synthesis of nanoparticles [5], electrochemistry [6,7] etc. Their application versatility arises from the number of possible combinations of parent substances (PS) needed for their syntheses. Unsurprisingly, they have also been investigated in various aspects of biomedicine, such as anti-bacterial treatment, drug delivery and solubilization.

The application of DESs has already been covered in many review articles [8,9,10,11,12], including two crucial applications in medicine—drug delivery enhancement and treatment of bacterial infections—for instance, El. Achkar et al. [12] summarized the different routes for synthesising DESs and the most relevant parameters controlling their formation and physicochemical properties. In a recent extended review article, Hansen et al. [10], after a brief discussion of the various application ranging from metallurgy to pharmaceutical and biomedical fields of DESs, gave a deep and detailed discussion of the literature results to understand the microscopic mechanisms governing the structure-property relationships. In the review report by Liu et al. [11], the importance of natural deep eutectic solvents (NADESs) in natural products and biological systems was highlighted through various applications, such as extraction and chromatographic media. Zainal-Abidin et al. [13] and Huang et al. [14] described respectively the application of DES in drug discovery and delivery systems and as active pharmaceutical ingredient (API) delivery systems in the treatment of metabolic diseases (cancer, diabetes, and atherosclerosis).

Moreover, using DES might be a better strategy than a straightforward transformation of APIs into ionic liquids (IL). First of all, not all of the APIs can be transformed into classic ILs—the targeted API should be in the ionic form that allows for the formation of IL. Common cations include imidazole derivatives or other *N*-heterocyclic compounds. Imidazole can sometimes be found in drugs (mainly in antifungal therapy [15]), but it is not a common structural motif. In addition, the size of the molecule is crucial for the formation of ILs. It is possible to form API-IL [16], but this usually applies to transforming small compounds, excluding biomacromolecules like peptides, proteins and modified DNA. Moreover, even though the development and medical-application optimization of ILs is ongoing, their cytotoxicity and environmental impact are still questioned [17,18,19]. Adding to it their high production cost and not-so-straightforward synthesis, it seems reasonable to look for alternative strategies for API delivery.

The study and application of DES is a very active field, as witnessed by the increasing number of reports over the last few years. Interestingly, many of the proposed DESs are composed of either carboxylic, (poly)carboxylic and/or fatty acids. The use of organic acids seems to be a very promising strategy, as carboxylic groups are easily involved in forming hydrogen bonding, which lies at the root of the formation of DESs. In addition, organic acids are already applied in medicine as drugs or antibacterial agents. Thus, it seems appealing to formulate them in the form of DESs and further investigate their properties.

In this review, we summarize the recent data on DESs that comprise organic acids. This report differs from the previous review articles in its content and focus. We discuss the developments of only DESs composed of organic acids and their applications in biomedicine (antimicrobial agents and drug delivery enhancers). First, to introduce the reader to why the choice of hydrogen bond donors/acceptors is so important from the application point-of-view, a theory behind DESs formation was discussed. This leads to a discussion of why classifying DES based on the type of parent substance (PS) used can help us better to evaluate their possible (bio)medical applications. The following section introduced a group of organic acid-based deep eutectic solvents (OA-DESs). The most interesting examples of their application, both as antimicrobial agents and drug delivery enhancers, were highlighted. This review’s primary purpose is to address whether OA-DESs are a suitable family of biomaterials for application in (bio)medicine.

### 1.2. Physicochemistry of Deep Eutectic Solvents (DESs)

**Figure 2:**
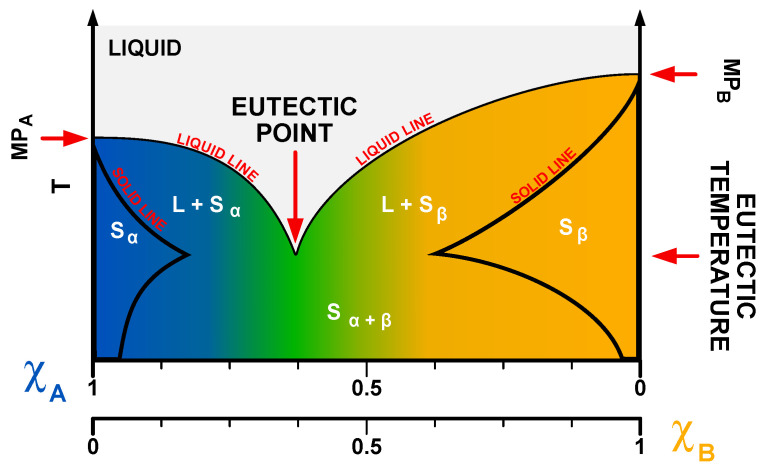
Phase diagram of the binary DESs. Note the presence of a deep eutectic point. S and L—solid and liquid phase, α and β—phase names, χ—molar fraction of substance A and B.

Deep eutectic solvents are mixtures of hydrogen bond donors (HBDs) and acceptors (HBAs) (Lewis and Brønsted acids and bases), which are characterized by the presence of the eutectic point. Eutectic point is associated with a specific ratio of parent substances (PSs) at which their mixture exhibits a much lower melting point than the isolated PSs. In the case of DESs, the depression of the melting point is much deeper than for common eutectics (Figure 2); hence, the term “deep eutectic”. An example of that phenomenon can be observed in the common DES called reline—a mixture of choline chloride (ChCl) and urea (U) in a molar ratio of 1:2, which was first discovered by Abbott et al. [1]. While the melting points of ChCl and U are 302–305 °C and 133–135 °C, respectively, the melting temperature of reline is ~12 °C.

Eutectic point occurs at a strict molar ratio of PSs, usually described by integers. This ratio is related to physicochemical interactions and structure of DESs and will be addressed later in this review. However, it should also be noted that certain DESs can still form a liquid at other PS ratios than the one at the eutectic point, and the eutectic point itself might not always be sharp. For this reason, the term eutectic temperature was introduced. Eutectic temperature can be defined as the lowest melting temperature of the eutectic throughout different ratios of PSs and is used interchangeably with the term ‘eutectic point’.

However, it has to be highlighted that there is still no clear understanding of how deep the melting point depression must be to define DES—or what should be the melting point of DES. It seems that the most common way of defining it, is by looking at the melting point of the mixture in the operational (applicational) temperature. Some authors assume DESs must be liquid below 100 °C [20]. Other ones state that they must maintain the liquid at room temperature [21]. We also know DESs with melting points as low as −40 °C (glycerine) [22]. However, there is no consensus between the researchers in the field, with more and more questions about the nature of DESs and the understanding of the relationship between their molecular structure and macroscopic properties [23]. For this work, we will adopt a definition which states that the eutectic mixture is considered *deep* when it is in a liquid state in the temperature range of 20–36 °C—which is sufficient for most biomedical applications.

**Figure 3:**
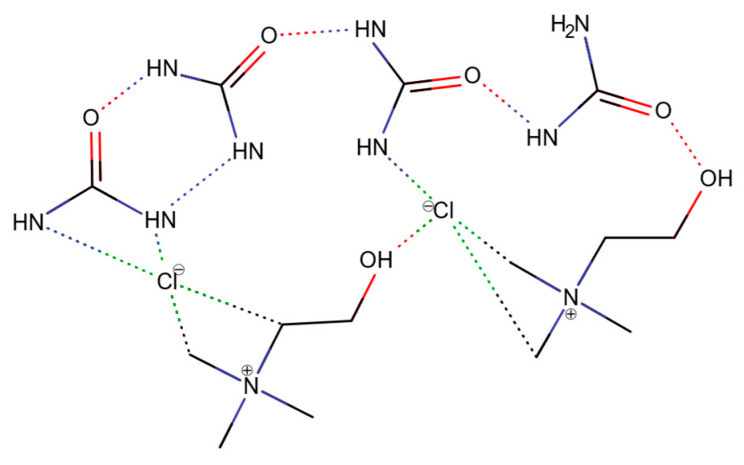
Schematic illustration of the interaction lattice between the molecules of reline (based on [25], © RSC).

The eutectic point of a DES is directly associated with its structure, which features a highly complex network of hydrogen bonds, resulting in specific physical properties such as low vapor pressure, high viscosity, and usually higher density than water. The direct evidence that the presence of the hydrogen bond lattice is responsible for the observed eutectic point was reported by Stefanovic et al. [24]. The mechanism of the formation of DESs has also been investigated by Araujo et al. on the example of reline, using inelastic neutron scattering [25]. In their study, they found that both choline chloride and urea undergo steric changes. The planar (sp^2^) structure of urea (in crystal) becomes tetragonal (sp^3^), and the chloride anion of ChCl is slightly displaced, which decreases the steric hindrance of the torsional movement of methyl groups. These two combined effects lead to the formation of the hydrogen bond lattice in the structure of reline. Therefore, it can be presumed that similar behavior is present in other organic DESs (Figure 3).

What has to be considered in the structural nature of DES is the way we describe the molecule of the DES itself. In 2021, Zhang et al. raised an interesting question—‘Should deep eutectic solvents be treated as a mixture of two components or as a pseudo-component?’ [26]. In the above-referenced work, the authors discussed how we should describe the molecular character of DESs. On the molecular level, are DESs mixtures of two components? Or should we perceive the molecules that build the DESs (and interact in the given molar ratio) like a single molecule, despite the lack of covalent bonds between parent substances? This factor is essential as it allows us to describe the molecular mass as M_DES_ = f_PS1_M_PS1_ + f_PS2_M_PS2_ (where f is a molar fraction, ∑f = 1) or as M_DES_ = yM_PS1_ + xM_PS2_ (where x and y are molar coefficients).

Based on viscosity, density, and spectral data of diluted ChCl-based DESs, they concluded that DESs exhibit features that reflect their pseudo-component nature. This is important for the proper description of the DESs interaction and concentration. It is worth noting that both molar and volume concentrations are reported throughout the published papers.

#### Current Categorization of DESs

DESs can be categorized in various ways, depending on the principles adopted. In one of the first classifications, based on the work by Abbott et al. [27], four categories of DESs were defined:

- TYPE I: quaternary ammonium salt + metal chloride
- TYPE II: quaternary ammonium salt + metal chloride hydrate (pseudo ternary system)
- TYPE III: quaternary ammonium salt + HBD
- TYPE IV: metal chloride hydrate + HBD

Alongside this classification, other terms describing particular families of DESs, were coined based on the origin of the PSs used and/or applications of DESs.

For example, therapeutic deep eutectic solvents (THEDESs) are DESs containing active pharmaceutical substances. The formation of DES either improves the efficacy of certain drugs and/or allows the material to be applied in other forms of therapy. THEDES formulation has many great benefits. One of them is the possibility of the penetration of the biological barriers. However, the most important one is API liquefaction, which helps overcome solubility limitations and polymorphism and improves drug bioavailability.

Some bright examples of THEDESs are mixtures of menthol with either ibuprofen (improvement of transdermal delivery) [28] and benzoic acid or phenylacetic acid (antimicrobial agents) [29,30]. Another example of THEDES studied in-depth is a choline chloride and geranic acid mixture. It worked nicely as an antimicrobial agent [31] and improved the transdermal delivery of insulin [32,33].

Another group of DESs are natural deep eutectic solvents (NADESs). NADESs may share some applications with THEDESs, but the criteria on which the family of these solvents emerged rely strictly on the PSs used. NADESs are synthesized using naturally occurring biomolecules—carbohydrates, amines, carboxylic acids etc. These molecules are common metabolites; therefore, NADESs are considered the greenest family of DESs. Good examples of this type of DESs are mixtures of glucose and sucrose as well as ChCl and maleic acid, which were used to improve the solubility of curcumin in photodynamic therapy [34]. NADESs can also be very good solubilizers. For example, NADES comprising malic acid, water, proline, and lactic acid enhanced the solubility of berberine (anti-inflammatory and antibacterial drug) almost ten times (25 mg/mL) when compared to water (2.10 mg/mL) or ethanol (2.75 mg/mL).

Deep eutectic solvent derivatives (DESDs) are another class of DESs. The definition of this group is not quite clear; however, a common feature of such mixtures is the lack of sharp eutectic points. Usually, such mixtures are ternary systems—like a mixture of ChCl, glycolic acid, and oxalic acid (OxA) [35]. However, it is worth noting that not much data is available about this particular group—especially regarding the proper physicochemical measurements that would allow us to verify the existence of a eutectic point.

As the above-mentioned classification systems are quite general, many DESs may (and usually do) overlap some of the definitions. Moreover, based on these systems, we cannot predict the biological and physical properties of DESs. This is where the categorization and analysis of DESs based on the type of PS in their application might be helpful, as the properties of DESs depend on the PS used.

The most common groups to form DESs are alcohols, (poly)alcohols, amines, carbohydrates, and carboxylic acids. The latter is particularly interesting due to their biological, chemical, and physical properties.

### 1.3. Organic Acids and Their Medicinal Properties

Carboxylic acids are a group of substances that exhibit many interesting chemical properties for their application in medicine. The presence of a carboxylic group endows the molecule with hydrophilicity. The pK_a_ of carboxylic groups of most of the organic acids is between 3.5 to 4.5; this means that the majority of these molecules will be in their deprotonated (ionized) form in biological media [36].

They usually bind to specific proteins/receptors, such as dopamine beta-hydroxylase (ascorbic acid [37]) or NimA-related protein (lactic acid [38]). Still, they are also involved in metabolic processes such as the Krebs cycle. A prime example of the interaction of organic acids with proteins is isobutylphenylpropionic acid, also known as ibuprofen (I). Its mechanism of action relies mainly on the reversible inhibition of COX-1 and COX-2 enzymes [39]. This widely used drug can migrate into the cell thanks to its partial solubility in lipids, facilitating its intake. It helps to relieve the pain while counteracting fever and inflammation. Ibuprofen is almost wholly metabolized with little or no trace in the urine.

**Figure 4:**
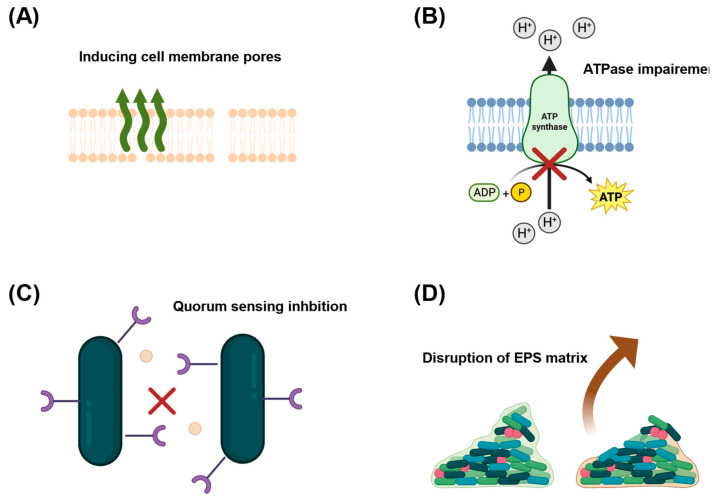
Mechanism of action of carboxylic acids on bacteria: (**A**) disruption of cell membrane structure, (**B**) ionic imbalance resulting in malfunction of various enzymes, (**C**) inhibition of QS pathways, (**D**) biofilm degradation by affecting extracellular polymeric substance (EPS) matrix.

Antibacterial properties of organic acids, such as acetic or benzoic acid, are also very intriguing (Figure 4). Some organic acids (mainly phenolic) can interact with the lipid layer of bacteria by dissolving in them and changing their charge and permeability [40,41]. This is due to other indirect effects, such as a change of polarity and/or disruption of the integrity of the cell membrane by forming pores. Also, an indirect interaction with specific proteins occur on the cell membrane. The cell membrane disruption leads to ion transport destabilization, resulting in the impairment of adenosine triphosphate (ATP) generating systems [42,43]. Quorum sensing (QS)—a mechanism of bacteria communication during which information about cellular density is exchanged, is of great importance for bacteria’s virulence and biofilms’ formation. Various organic acids can counteract the growth of bacteria through interaction with QS either by inhibiting the synthesis, functionality, and transport of autoinducers or by antagonizing autoinducer receptors [44,45]. Thanks to this mechanism, adhesion and maturation of biofilm are also inhibited [46].

**Figure 5:**
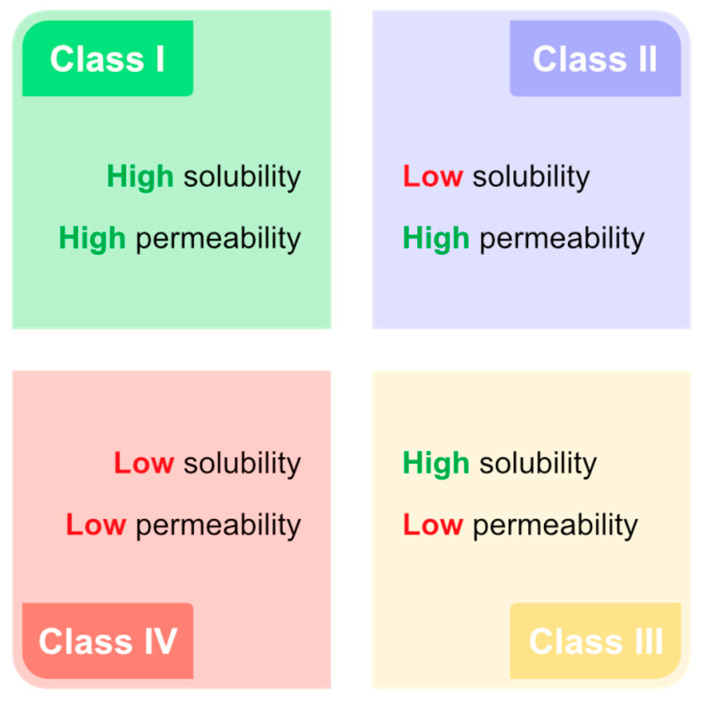
Biopharmaceutical classification system of drugs.

Due to their structure, which allows hydrogen bond network formation, organic acids can be used as HBDs/HBAs to form organic acid-based DESs (OA-DESs). Such formulations are expected to be water soluble and combine desired features of organic acids while increasing their permeability—placing OA-DESs as a class I drug, according to the biopharmaceutical characterization system of drugs (Figure 5).

## 2. OA-DESs Applications in Biomedicine

### 2.1. Antibacterial Properties of OA-DESs

As many organic acids exhibit antibacterial properties, it seems rational to try to improve their efficiency through their formulation as DESs, given that the DESs physical properties (high viscosity and low vapor pressure) may prolong their interaction with tissues. This should allow OA-DESs, for example, to exhibit a synergistic effect on biofilms and the possibility of controlled API release if needed.

**Figure 6:**
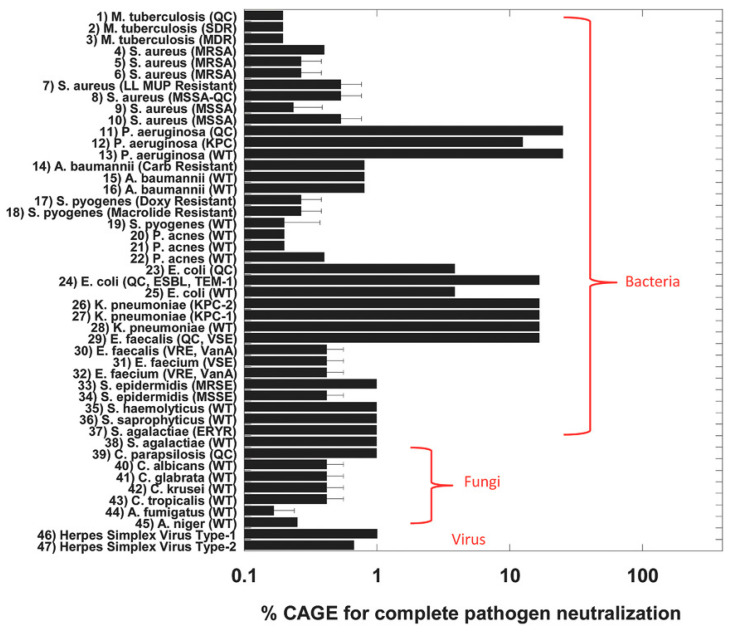
The effectiveness of CAGE OA-DES against 47 pathogens, including bacteria, viruses, and fungi ([31], © Wiley).

A prime example of the antimicrobial properties of OA-DESs was reported in 2016 by Zakrewsky et al. [31], who investigated a mixture of geranic acid and choline bicarbonate (CAGE) in a 2:1 molar ratio. CAGE has been tested on 47 pathogens, including bacteria, fungi, and viruses (Figure 6). It displayed excellent antimicrobial properties while being benign to human cells and tissues. Geranic acid does not mix with water. However, after heating together with water-soluble choline bicarbonate, it could form a stable mixture. CAGE has been shown to have extremely low MBC of around 0.2–0.5% for *M. tuberculosis*, Methicillin-resistant *S. aureus* (MRSA), *P. acnes*, *C. albicans* and *A. niger*. In addition, it was also very effective against Herpes Simplex Virus (HSV) Type-1 and 2 (Table 1). Based on dynamic light scattering (DLS) analysis, it was suggested that the possible mechanism of bacterial inhibition occurs through their inactivation rather than destruction by OA-DES.

CAGE has been tested with benzalkonium chloride (commonly found in disinfectants), povidone-iodine and chlorhexidine acetate (presurgical sanitizer) for its cytotoxicity against keratinocytes. CAGE maintained almost 100% cell viability up to 10 mM, while referral substances killed practically 100% of the cells at concentrations as low as 0.01 mM. This result highlights that this OA-DES is an excellent candidate for a new generation of topical partially hydrophobic disinfectants [31].

Regarding limited hydrophilicity, a fascinating group of organic acids is saturated fatty acids. They share some of the properties of simple carboxylic acids. Still, the presence of saturated carbon chains makes them partially dissolve in lipids, which might be a key to the penetration of the biological membranes. Saturated fatty acids can be used to synthesise DESs; however, during microbiological tests, the factor of diffusion of DESs in the medium must be considered. DESs are liquids, and due to diffusion-related phenomena when compared to solid substances like many antibiotics and disinfectants, they should be investigated by using disk-diffusion assays and assessing their MIC/MBC. In addition, in the case of fatty acids, which do not dissolve in water, a small amount of DMSO has to be used to optimize the measurements and improve their solubility while limiting the cytotoxicity towards healthy (human) cells [47].

Combining fatty acids with APIs is possible, which results in THEDES. As an example, menthol (M), which is non-soluble in water (0.4 mg/L), is capable of forming stable DESs with stearic (SteA), lauric (LauA) and myristic (MyrA) acids [48]. Further investigation revealed that a mixture of M:SteA exhibited the lowest cytotoxicity towards HaCaT cells. Stearic acid showed no MIC/MBC due to a complete lack of solubility, while MIC and MBC values of menthol were between 4–8 and 8–16 mM, respectively (depending on the strain investigated (*S. aureus*, MRSA, MRSE)). After solubilization in the form of DESs, the MIC decreased to around 3.52–6.52 mM and MBC to 6.52–13.03 mM. Interestingly, DESs of M: SteA have been proven to boost the wound healing process by increasing the recovery of wound area by around 20% compared to control.

**Figure 7:**
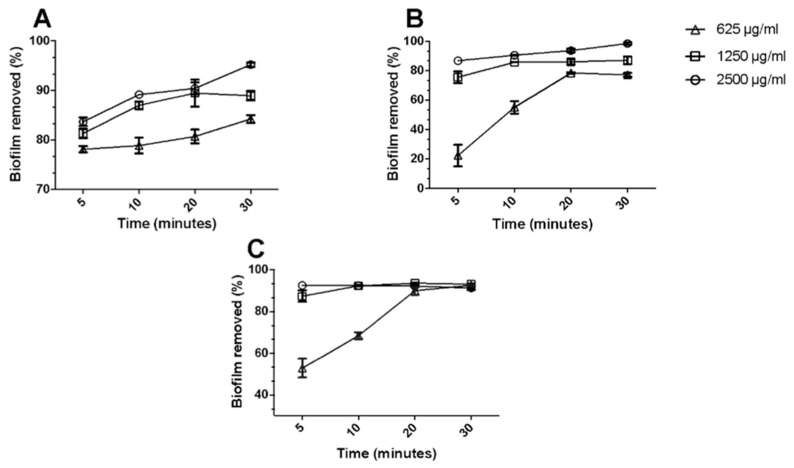
Percentage of biofilm removal upon exposure to different concentrations of CapA:LauA (625, 1250 and 2500 μg/mL) for a total period of 30 min for (**A**) *E. coli*, (**B**) MRSA and (**C**) *C. albicans* ([47], © Elsevier).

The same fatty acids, mixed as binary OA-DESs with capric acid (CapA), have been tested for bacterial eradication and biofilm removal potential [47]. While none of the OA-DESs inhibited the growth of Gram-negative *E. coli* and *P. aeruginosa*, they were effective against Gram-positive *S. aureus* (MRSA), MRSE and yeast strains of *C. albicans*. Such behavior is not surprising as fatty acids’ antibacterial effect might be hindered by the presence of lipopolysaccharides [52,53]. Based on the initial results, a mixture of capric acid and lauric acid (CapA:LauA) has been chosen to study the effectiveness of biofilm removal of *E. coli*, MRSA and *C. albicans*. Interestingly, CapA:LauA was able to eliminate around 80–90% of all the biofilms within 20 min., with more than 50% of the biofilm removal in the first 5 min (Figure 7). These results indicate that fatty-acid-based OA-DESs might comprise two effects: antibacterial properties (observed by the presence of inhibition halos and MICs/MBCs) and biofilm removal potential. The second effect might result from the dissolution of biofilm components in DESs. Such behavior is justified as a biofilm matrix comprising lipids, extracellular DNA, EPS, and extracellular vesicles [54,55].

When considering fatty acid-based OA-DESs, one realizes how broad the definition of DESs has become. As a result, mixtures such as those mentioned above can no longer be classified using the classical quadruple division proposed by Abbott et al. This also highlights the importance of thorough thermal and microscopic (POM) investigation of novel mixtures to prove that eutectic point exists.

Fatty acids seem to be very effective against Gram-positive bacteria. However, most dermal infections are caused by the formation of biofilms composed of Gram-negative and Gram-positive bacteria. Therefore, to increase the effectiveness of OA-DESs, alternative approaches have been studied. Following the idea of CAGE, where much smaller molecules are used, formulations that comprised menthol mixed with phenylacetic (PhaA), acetylsalicylic (AcA) or benzoic (BenzA) acid have been studied as well [30]. These substances have been proven effective antimicrobial agents [56,57,58,59,60,61]; thus, their DESs formulations have been assessed against *E. coli*, *S. aureus* and *B. subtilis*. In their approach, Aroso et al. [30] applied the above-mentioned acids as APIs, whose effects have been boosted by menthol. All the formulations have been effective against all the tested strains with MIC as slow as 1 mg/mL for *B. subtilis* and as high as 4 mg/mL for Gram-negative *E. coli*.

In the case of DESDs, ternary systems comprising organic acids have also been tested. One of the examples is the OA-DES of betaine (B), malic acid (MalA) and glucose (G) or proline (P) in a 1:1:1 ratio [49]. Betaine acts as an HBA, while malic acid and glucose or proline are HBDs. This mixture seems to be an excellent formulation due to the use of small metabolites expected to exhibit low cytotoxicity and good biocompatibility. The formulations were effective against *E. coli*, *P. mirabilis*, *S. Typhimurium*, *P. aeruginosa* and *S. aureus*. In the same study, the authors demonstrated that OA-DESs that comprised citric acid (together with glucose or fructose and glycerol) were even more effective towards the same strains of bacteria. It also seems that using small metabolites as PSs affected the EC_50_ values towards HeLa, HEK293T, and MCF-7 cells with values over 2 mg/mL. The joint effect of high antibacterial potential with low cytotoxicity makes such combinations even more promising materials, which may be looked into in the near future.

Based on the examples above, we can state that OA-DESs own great potential as active antimicrobial agents. Not only do they manage to kill Gram-positive effectively, but also Gram-negative bacteria. The latter is particularly interesting due to another double-lipid membrane and periplasmic space, which inhibit most substances from penetrating their cytoplasm. Also, as evidenced for fatty acid-based OA-DESs, particular specificity may be reached based on the presence of the Gram layer. OA-DESs have proven to be also effective against MRSA, MRSE and HSV—pathogens being hard to neutralize. This makes OA-DESs a promising alternative to small-molecule antibiotics. Even though not every reported OA-DES is soluble in water (i.e., ones that comprised menthol)—they can also be applied topically for the potential removal of biofilms, which should enhance the process of wound healing. Overall, OA-DESs are a group of materials which will be developed further in this direction, even though more testing for their cytotoxicity towards healthy human cells is required. They act as antibiotics while also having protein extraction potential, which is rarely tested ex vivo.

### 2.2. Drug Delivery Using OA-DESs

Another valuable aspect of medicine is drug delivery. However, drug delivery often encounters the problem of low bioavailability, which could be due to several factors:

- route of administration
- permeability
- solubility
- stability

There are different ways of tackling these challenges. In general, one can either change the route of administration (if possible), chemically modify the drug to enhance its solubility, and/or encapsulate it to improve its solubility and stability. Different strategies have been proposed over the recent years, but the general target is the same, i.e., achieve the lowest possible final drug concentration, which still ensures its effectiveness. That is why technologies targeted at improving drug permeability while increasing solubility and maintaining stability are crucial to resolving this hurdle. Here, OA-DESs might be a handy tool to ensure all these aspects. As OA-DESs function as microenvironments, they provide an elastic hydrogen bond lattice able to stabilize most of the molecules. Furthermore, depending on the composition of OA-DESs, one can alter the hydrophilicity of the solvent, enabling more significant dissolution of drugs that poorly dissolve in water. The high viscosity of OA-DESss also limits the contact of the API with oxygen, which prevents it from atmospheric oxidation.

The first ever work on the use of DESs in the delivery of API was reported in 1998 by Stott et al. The authors proposed OA-DESs, composed of ibuprofen and terpenes: ld-menthol, l-menthol, thymol, and 1,8-cineole [28]. By measuring the flux of ibuprofen, they found that all the investigated terpenes enhanced the flux of ibuprofen, with thymol-driven enhancement by 11 times. This work sparked interest in transdermal delivery enhancement by DESs—mainly, that organic acids seemed to be excellent HBDs.

Following that, transdermal delivery of ibuprofen using OA-DES was reported in 2015 by Park and Prausnitz [62]. Their study demonstrated the effectiveness of ibuprofen/lidocaine DES in vivo as an anesthetic. In addition, they found out that the solubilization of lidocaine with ibuprofen affected its permeability and increased the absorption of lidocaine in rat models. Another example of the application of ibuprofen as PS of OA-DES was described in 2019 by Pereira et al. [63]. They have investigated four formulations of limonene with menthol, capric acid or ibuprofen. All the OA-DESs were effective antiproliferative agents, even though only OA-DES of ibuprofen and limonene (in 1:4 ratio) inhibited HT29 proliferation without comprising cell viability, inferring that this formulation is a potential anti-cancer drug and similar formulations may be developed soon, either as a direct use as a drug or as a medium for synthesis of other drugs [64].

In the case of drug delivery, non-steroidal anti-inflammatory drugs (NSAIDs) are also of particular interest. Such drugs as ibuprofen, acetaminophen or naproxen have poor solubility in water, and thus their effect may be inhibited when administered in aqueous solutions. Here, OA-DESs may be a viable means, as they can improve the solubility of different substances. Formulations proposed by Lu et al. [65] seem to be promising. Mixtures of different HBAs/HBDs proved very effective when solubilizing NSAIDs. Among the 18 tested formulations, 10 of them comprised organic acids. For HBAs, choline chloride, choline bitartrate (ChBT), betaine, tetrapropylammonium bromide (TPAB) and ethylammonium chloride (EACl) were investigated. At the same time, malonic (MaloA), oxalic acid, levulinic acid (LevuA), lactic acid (LacA), glutaric acid (GluA), and glycolic acid (GlycA) have been tested. Stable OA-DESs have been assessed as solvents for five NSAIDs: aspirin, naproxen, ibuprofen, ketoprofen and acetaminophen (Table 2). The results were impressive—thanks to the use of OA-DESs, the solubility of aspirin and acetaminophen was increased 5 to 20 times. As for the poorly soluble drugs (ibuprofen/naproxen/ketoprofen), OA-DESs improved their solubility by 4000 times. As the type of HBD seems to play a significant role in the solubility of API, HBA will also affect this. A comparison of OA-DESs comprising levulinic acid revealed that switching ChCl for other HBA improved the solubility of all the drugs, especially those poorly dissolving in water.

It is worth noting here that OA-DESs have also been applied as effective antibiotic carriers. In their original work, Zakrewsky et al. [31] used CAGE to treat ear infections in vivo on a rat model. When compared with saline, CAGE itself was much more effective than 1% solution of clindamycin. However, combining the two reduced rat ear thickness by ca. 70% after four days, compared to the eight days required for pure CAGE. This indicates that CAGE successfully boosted the antibacterial effect of the antibiotic by improving its transdermal delivery.

However, the inner dissolution of substances is also an important criterion when it comes to the synthesis of DESs. Based on the work of Aroso et al. [30], the dissolution efficiency of three APIs—PhaA, AcA and BenzA increased when combined with menthol to form a THEDES (Table 3). PhaA, AcA and BenzA have been previously investigated for their antimicrobial properties and proved to be effective agents against fungi and bacteria [66,67,68]. Interestingly, MIC and MBC values of the tested DESs were higher than those of pure PSs. This indicates that, even though the dissolution of the APIs has increased, their effect has been slightly hindered. In contrast, similar studies by Duarte et al. [29] showed that OA-DES formulations of ibuprofen, benzoic acid and phenylacetic acid with menthol had much better permeability and diffusion coefficient when compared to neat parent substances (Table 3).

High permeability and a high diffusion coefficient resulted in much lower diffusion times through polyethersulfone membranes, making this type of OA-DESs a very promising delivery system. As the influence of the structure of HBA/HBD was not so often discussed, the authors used NMR to assess how the structure of organic acid affects diffusion and found that the size of the API molecule does not influence its diffusion [29].

OA-DESs and DESs are good solvents for the dissolution of small molecules and for much bigger, high-molecular-weight biomolecules. Here, one of the first studies conducted in 2013 by Choi et al. [69] demonstrated that DNA (from male salmon), albumin, and amylase displayed good solubility in certain NADESs, with DNA having 46% higher solubility in OA-DESs consisting of malic acid and proline when compared to water. Following this work, Dai et al. [70] assessed more than 70 NADESs for solubilizing small biomacromolecules. In the final group of 5 tested NADESs, OA-DES of lactic acid, glucose, and water seemed particularly effective in dissolving gluten (88 times more effective) and DNA (34 times more effective). Moreover, the same OA-DESs enabled the dissolution of starch up to a concentration of 7.5 mM.

Proteins themselves are interesting components in the aspect of their delivery. However, their structure-dependent function relies on many weak interactions, namely hydrogen bonds, hydrophobic interactions, π-π stacking, and electrostatic interactions (including van der Waals forces) [71,72]. This makes their function susceptible to environmental changes, often limiting their availability and solubility. Here, OA-DESs, characterized by highly developed hydrogen bond lattice and elasticity of molecules with high coordination potential, might be interesting. Choi et al. [69] first raised awareness of the role of NADESs, naturally found in living organisms, on the stability of proteins. This hypothesis was reevaluated in 2013 by Esquembre et al. [73], in 2015 by Su and Klibanov [74], and in 2017 by Sanchez-Fernandes et al. [75] using model proteins and demonstrated that DESs are well-structured and activity preserving solvents.

Parallel to the studies mentioned above, applications of choline-based OA-DESs in the dissolution and transport of certain proteins have been conducted (Table 4). In 2018, Tanner et al. [33] delivered transdermal insulin using CAGE and its variants. Formulations of 1:1, 1:2, 1:4 and 2:1 of CAGE have been investigated for their conductivity and viscosity, thermal stability as well as by NMR to characterize the internal interactions of solvents. In their research, the authors first studied the interactions of CAGE formulations on the *stratum corneum* (outermost layer of skin) to characterize the possible transport mechanism. As revealed by FT-IR spectroscopy, due to its partial hydrophobicity, CAGE acts as a solvent for lipids, disrupting lipid bilayers. Moreover, the extraction potential strictly depends on the amount of geranic acid in CAGE, with a 1:4 ratio being as effective as the neat geranic acid itself. Interestingly, though the 1:2 and 1:4 mixtures were the most effective in transporting insulin (as verified by confocal microscopy using diffusion Franz cell), the effectiveness of other ratios was comparable to PBS.

Furthermore, OA-DESs have also been used to transport biomolecules such as monoclonal antibodies. These specific antibodies are currently used in the therapy of (among others) cancer [77,78,79] and arthritis [80,81]. They are usually administered via subcutaneous or intravenous injection, but OA-DESs might open a possibility for their delivery via the gastrointestinal tract. In their recent work, Angsantikul et al. [76] studied the effectiveness of a mixture of choline chloride and glycolic acid (“CGLY”, in 2:1, 1:1 and 1:2 molar ratios) in the transport of TNFα antibody, focusing on the stability of the antibody in OA-DESs, in vitro transport and in vivo uptake. Interestingly, CGLY (2:1 and 1:1 ratios) had a negligible effect on the stability of the antibodies, which have retained their high binding activity even at 20% *v*/*v* of saline. At the same time, the 1:2 mixture inhibited the binding almost wholly. CGLY 2:1 and 1:1 did not affect the functionality of TNFα when stored at 4 °C; however, the 2:1 ratio inhibited the binding ability of the antibody when stored at room temperature. Cell viability on the Caco-2 cell line resulted in ratio-dependent IC_50_—the smaller the fraction of choline bicarbonate, the lower the IC_50_. The ratio of 2:1 turned out to be the best candidate and was tested further. The final results have underlined that CGLY 2:1 was an excellent transporter of the antibody, exerting no adverse effect on the rat model. Interestingly, thanks to its micro-extraction potential, this OA-DES decreased the viscosity of mucus, which enhanced the antibody uptake.

An interesting formulation of OA-DES was also proposed by Haraźna et al. [82]. They have investigated bacteria-derived hydroxy fatty acids HFA as HBDs, while three ammonium salts, namely choline chloride, tetrabutylammoniummethyl chloride (TBMACl), and 1-ethyl-3-methylimidazolium chloride (EMlmCl) served as HBAs. The formulated OA-DESs have been compared with similar DESs where, instead of a mixture of HFAs acids, a mixture of aliphatic nonanoic and heptanoic acids (FA) was used (Table 5). Obtained data suggested an HBA-dependent solubility of lignin in the investigated OA-DESs. EMlmCl seemed to be the best HBA in the investigated formulations, as the solubility of lignin increased 100 times (up to 30 mg/mL) compared to neat PS. On the other hand, the efficiency of TBMACl was much lower, and for HFA-based OA-DESs, it was only 2–3 times higher than that of PS and 7 and 15 times higher when FA was used. Surprisingly, ChCl was the least effective as an HBA, and lignin dissolution was only observed for one of the four investigated OA-DESs.

Looking at all the examples above, we can clearly state that OA-DESs significantly impact the dissolution of drugs. Not only do they improve the solubility of small molecular drugs, but they can also act as APIs themselves. Owing to their physicochemical properties, they seem to be an excellent environment for the dissolution of high molecular weight molecules such as proteins and peptides—a challenge we still face today. It must be pointed out that even though using OA-DESs increases the solubility of certain APIs, reaching therapeutic concentrations is still a challenge. Here again, we must look at the main factors limiting drug bioavailability. If by implementing OA-DESs, we can increase the solubility and stability of the API for certain routes of administration. More drugs will reach the target. If more drugs will be delivered using OA-DESs compared to other administration strategies, then adaptation of OA-DESs is beneficial and a frog leap in this field. Hopefully, in the following years, we will see a clear development in this topic and, finally, a successful application in vivo.

## 3. Perspectives and Challenges to Overcome in the Future

Even though OA-DESs seem to be a very promising family of materials, more research is needed to assess their biological properties properly. As antimicrobial agents, we can observe differences between disk-diffusion assays and MIC/MBC tests, which probably arise from the liquid state of DESs and diffusion-correlated behavior on the surface of agar plates. The chimeric properties of OA-DESs are reflected in the MIC/MBC values, which are usually lower for OA-DESs when compared with parent substances (PS). This indicates different behaviors of OA-DESs on surfaces than in the solution. In addition, the efficacy of OA-DESs for biofilm removal and eradication deserves much attention because biofilms are recalcitrant to antibiotic therapy and represent a significant cause of persistent and recurrent infection by clinically essential pathogens. In this line, the combination of OA-DESs with known antibiotics or other antimicrobial molecules is expected to overcome the resistance exhibited by bacteria for the most common antibiotics, owing to the high micro-extraction power of DESs.

OA-DESs proved to be an interesting strategy in dissolving active pharmaceutical ingredients (API) either as PS of OA-DESs itself or as a separate component. As DESs polarity and internal interactions can be manipulated by the choice of proper PS, they represent an excellent environment for the dissolution of small molecules (NSAIDs, antibiotics) and macromolecules like proteins and DNA. Here, the hydrogen bond formation potential that arises from carboxylic groups, present in the structure of organic acids, seems to play a crucial role in this process. The presence of other hydrogen bond donor groups is not affecting the solubility of API to the same extent as the carboxylic group; however, they influence other parameters, such as melting point and/or viscosity, which are also equally important for materials designated for medical use. Although the efficacy of OA-DESs in improving drug solubility and bioavailability has been demonstrated in many papers, applications of DESs for sustained drug release are still to be demonstrated. Moreover, for implementing DESs in drug delivery systems, their toxicological profiles and biosafety in vivo should be assessed.

Regarding new formulations, the number of possible combinations of PSs that form DESs seems to be limited by the availability of precursor components. This poses a significant problem in synthesis focused on specific materials’ applications. Furthermore, even within specific groups of HBAs/HBDs, the physicochemical and biological properties of DESs vary. As for now, mainly a strategy of trial and error is implemented with only a few attempts using theoretical methods. Another possible approach for predicting the properties of OA-DESs and DESs, in general, is using chemometrical methods to create a database of possible combinations of PSs, which will correlate with desired properties of DESs. However, even if this problem seems more pronounced, OA-DESs have already proven to be excellent biomaterials for medical use. Hopefully, they will be more developed in the future.

There seem to be still a lot of challenges for OA-DESs to overcome to be adapted on a larger scale in medicine. Due to many possible combinations of parent substances, more application-focused research strategies must be implemented. In the current state of biomedical sciences, we have to investigate such materials on a much broader scale than before, including thorough physicochemical characterization, assessment of their antimicrobial properties on more complex models (biofilms, ex vivo, in vivo), as well as their cytotoxicity.

## 4. Summary

Organic-acid-based deep eutectic solvents (OA-DESs) are an interesting class of biomaterials. Owing to their specific properties, such as antimicrobial properties, low cytotoxicity towards mammalian cells and tissues, the potential to be a class I drug and being a good (and green) solvent for many other drugs, they can be applied in many aspects of biomedicine. However, as the field seems to be slightly hindered by the number of possible strategies for synthesising DESs, we hope that organic acid-based DESs will be more and more investigated, enabling further research development in the biomedical application of DESs.

## Figures and Tables

**Table 1:** Antibacterial activity (and minimum inhibitory values (MIC)) of chosen OA-DESs against selected bacteria and yeast.

	Gram-Negative				Gram-Positive					Yeast	Ref.
	*S. typhimurium*	*P. aeruginosa*	*P. mirabilis*	*E. coli*	*B. subtilis*	*S. aureus*	MRSA	MRSE	*P. acnes*	*C. albicans*	
CapA:LauA	N/I	−	N/I	−	N/I	+ (625)	+ (625)	+ (625)	N/I	+ (625)	[47]
CapA:MyrA	N/I	−	N/I	−	N/I	+ (625)	+ (625)	+ (625)	N/I	+ (625)	[47]
CapA:SteA	N/I	−	N/I	−	N/I	+ (1250)	+ (1250)	+ (1250)	N/I	+ (1250)	[47]
M:SteA	N/I	N/I	N/I	N/I	N/I	+ (1018)	+ (2036)	+ (2036)	N/I	N/I	[48]
CAGE	N/I	+	+	+	N/I	N/I	+	+	+	+	[31]
B:MalA:P	+	+	+	+	N/I	+	N/I	N/I	N/I	N/I	[49]
B:MalA:G	+	+	+	+	N/I	+	N/I	N/I	N/I	N/I	[49]
M:BenzA	N/I	N/I	N/I	+ (4000)	+ (1000)	+ (2000)	N/I	N/I	N/I	N/I	[30]
M:PhaA	N/I	N/I	N/I	+ (4000)	+ (2000)	+ (2000)	N/I	N/I	N/I	N/I	[30]
M:AcA	N/I	N/I	N/I	+ (4000)	+ (2000)	+ (2000)	N/I	N/I	N/I	N/I	[30]
ChCl:OxA	+	+	+	+	N/I	+	N/I	N/I	N/I	+	[30]
Reline	+	+	+	+	N/I	+	N/I	N/I	N/I	N/I	[30]
CitA:P	+	+	+	+	N/I	+	N/I	N/I	N/I	N/I	[30]
CitA:Gly:G	+	+	+	+	N/I	+	N/I	N/I	N/I	N/I	[30]
CitA:F:Gly	+	+	+	+	N/I	+	N/I	N/I	N/I	N/I	[30]
CapA:M:Solutol	N/I	−	N/I	+ (25)	+ (0.1)	+ (0.1)	N/I	N/I	N/I	+	[50]
CapA:M	N/I	N/I	N/I	+ (1120)	N/I	+ (2240)	N/I	N/I	N/I	N/I	[51]
CapA:T	N/I	N/I	N/I	+ (1140)	N/I	+ (1140)	N/I	N/I	N/I	N/I	[51]

“+”—strain susceptible to OA-DES, “−”—strain non-susceptible to OA-DES, “N/I”—strain not investigated for susceptibility; MIC (if reported) was given in parentheses (µg/mL).

**Table 2:** Solubility of chosen NSAIDs in water and examined OA-DESs (adapted with permission from [65]).

	Aspirin	f	Acetaminophen	f	Ketoprofen	f	Naproxen	f	Ibuprofen	f
WATER	7.03 ± 0.03	-	19.95 ± 0.12	-	0.34 ± 0.00	-	0.06 ± 0.00	-	0.07 ± 0.00	-
ChCl:GluA	76.28 ± 0.61	10.85	238.10 ± 3.73	11.93	60.32 ± 0.42	177.41	21.04 ± 0.33	350.67	25.38 ± 0.20	362.57
ChCl:GlycA	67.43 ± 1.42	9.59	224.80 ± 3.02	11.27	22.09 ± 0.19	64.97	8.37 ± 0.09	139.5	9.49 ± 0.05	135.57
ChCl:MaloA	126.30 ± 2.06	17.96	266.3 ± 2.44	13.35	60.99 ± 0.31	179.38	21.98 ± 0.21	366.33	25.87 ± 0.13	369.57
ChCl:OxA	86.32 ± 1.16	12.28	186.70 ± 1.49	9.36	8.53 ± 0.21	25.09	13.90 ± 0.13	231.67	31.51 ± 0.20	450.14
ChCl:LevuA	135.00 ± 0.19	19.2	279.00 ± 3.20	13.98	192.90 ± 3.62	567.35	31.67 ± 0.40	527.83	78.69 ± 0.93	1124.14
ChCl:LacA	91.30 ± 1.83	12.99	254.00 ± 1.84	12.73	38.88 ± 0.53	114.35	12.95 ± 0.07	215.83	17.95 ± 0.25	256.43
EACl:LacA	44.57 ± 0.71	6.34	141.20 ± 0.73	7.08	32.24 ± 0.18	94.82	3.58 ± 0.04	59.67	20.67 ± 0.47	295.29
TPAB:LevuA	163.70 ± 2.30	23.29	316.50 ± 1.94	15.86	299.80 ± 3.33	881.76	129.10 ± 0.83	2151.67	194.80 ± 1.57	2782.86
B:LevuA	149.80 ± 0.96	21.31	212.10 ± 1.92	10.63	329.10 ± 4.42	967.94	76.64 ± 0.72	1277.33	280.20 ± 2.68	4002.86
ChBT:LevuA	37.06 ± 0.51	5.27	89.07 ± 1.04	4.46	51.02 ± 0.64	150.06	9.45 ± 0.11	157.5	35.05 ± 0.42	500.71

“f”—dissolution improvement factor (*f-times* increase) of a drug in OA-DESs compared to water.

**Table 3:** Dissolution efficiency of AcA, PhaA and BenzA in phosphate buffer saline (PBS) and formulated in DESs (adapted with permission from [29,30]).

PS/OA-DES	Dissolution Efficiency (%)	Permeability (10^5^ cm s^−1^)	Diffusion Coefficient (10^6^ cm^2^ s^−1^)	Time * (h)
AcA	11	N/I	N/I	N/I
M:AcA (3:1)	72	N/I	N/I	N/I
BenzA	70	0.9 ± 0.01	0.73 ± 0.09	3.5
M:BenzA (3:1)	87	6.8 ± 0.63	6.43 ± 1.42	0.5
PhaA	37	16 ± 2.3	3.14 ± 0.18	2
M:PhaA (3:1)	81	18 ± 0.38	4.66 ± 0.13	3
M:PhaA (2:1)	78	13 ± 0.59	5.73 ± 0.43	2
I	N/I	4.6 ± 0.14	2.39 ± 0.43	>8
M:I (3:1)	N/I	14 ± 1.53	4.32 ± 0.34	3

* time needed for 50% release of the API through polyethersulfone membrane.

**Table 4:** Selected OA-DESs as protein carriers.

OA-DES	Protein	Reference
CAGE	BVA	[32]
CAGE	OVA	[32]
CGLY	TNFα	[32]
CAGE	INS	[32,33]
CAGE	GLP-1	[76]

“BVA”—bovine serum albumin, “OVA”—ovalbumin, “INS”—insulin, “GLP-1”—glucagon-like peptide-1.

**Table 5:** Solubility of lignin in bacteria-derived OA-DESs and HFA-based OA-DESs (adapted with permission from [82]).

OA-DES/PS	Lignin Solubility (mg/mL)
HFA:TBMACl (1:1)	0.6
HFA:TBMACl (2:1)	0.9
HFA:EMlmCl (1:1)	0.8
HFA:EMlmCl (2:1)	26.9
HFA:ChCl (1:1)	-
HFA:ChCl (2:1)	25.6
FA:TBMACl (1:1)	7.2
FA:TBMACl (2:1)	15.6
FA:EMlmCl (1:1)	29.8
FA:EMlmCl (2:1)	30.7
FA:ChCl (1:1)	-
FA:ChCl (2:1)	-
HFA	0.3
FA	0.2

## Data Availability

All the data mentioned in this work are available under their identifiers that can be found in the “references” section. No new data were created, so data sharing is not applicable to this article.

## Abbreviations

AcA	acetylsalicylic acid
API	active pharmaceutical ingredient
ATP	adenosine 5′-triphosphate
BenzA	benzoic acid
B	betaine
CapA	capric acid
CAGE	deep eutectic solvent composed of choline bicarbonate/geranic acid
CGLY	deep eutectic solvent composed of choline bicarbonate/glycolic acid
ChCl	choline chloride
ChBT	choline bitartate
CitA	citric acid
COX-1/2	cyclooxygenase 1/2
DESD	derived deep eutectic solvent
DES	deep eutectic solvent
DLS	dynamic light scattering
DMSO	dimethylsulfoxide
DNA	deoxyribonucleic acid
EACl	ethylammonium chloride
EC50	half maximal effective concentration
EPS	extracellular polymeric substances
F	fructose
FA	mixture of nonanoic and heptanoic acid
FT-IR	Fourier transform infrared spectroscopy
G	glucose
GluA	glutaric acid
Gly	glycerol
GlycA	glycolic acid
HaCaT	immortalized human keratinocytes
HBA	hydrogen bond acceptor
HBD	hydrogen bond donor
HFA	bacteria-derived hydroxy fatty acids
HSV	herpes simplex virus
I	ibuprofen
IL	ionic liquid
LacA	lactic acid
LauA	lauric acid
LevuA	levulinic acid
MyrA	myristic acid
M	menthol
MalA	maleic acid
MaloA	malonic acid
MBC	minimum bactericidal concentration
MIC	minimum inhibitory concentration
MP	melting point
MRSA	methicillin-resistant Staphylococcus aureus
MRSE	methicillin-resistant Staphylococcus epidermidis
NADES	natural deep eutectic solvent
NMR	nucleic magnetic resonance spectroscopy
OxA	oxalic acid
OA-DES	organic acid-based deep eutectic solvent
P	proline
PhaA	phenylacetic acid
PS	parent substance
QS	quorum sensing
SteA	stearic acid
T	thymol
TE	eutectic temperature
THEDES	therapeutic deep eutectic solvent
TPAB	tetrapropylammonium bromide
U	urea

## References

[1] Abbott A.P., Capper G., Davies D.L., Rasheed R.K., Tambyrajah V. (2003). Novel Solvent Properties of Choline Chloride/Urea Mixtures. Chem. Commun..

[2] Vian M., Breil C., Vernes L., Chaabani E., Chemat F. (2017). Green Solvents for Sample Preparation in Analytical Chemistry. Curr. Opin. Green Sustain. Chem..

[3] Ünlü A.E., Arıkaya A., Takaç S. (2019). Use of Deep Eutectic Solvents as Catalyst: A Mini-Review. Green Process. Synth..

[4] Smith E.L. (2013). Deep Eutectic Solvents (DESs) and the Metal Finishing Industry: Where Are They Now?. Trans. Inst. Met. Finish..

[5] Liao H.-G., Jiang Y.-X., Zhou Z.-Y., Chen S.-P., Sun S.-G. (2008). Shape-Controlled Synthesis of Gold Nanoparticles in Deep Eutectic Solvents for Studies of Structure–Functionality Relationships in Electrocatalysis. Angew. Chem. Int. Ed..

[6] Figueiredo M., Gomes C., Costa R., Martins A., Pereira C.M., Silva F. (2009). Differential Capacity of a Deep Eutectic Solvent Based on Choline Chloride and Glycerol on Solid Electrodes. Electrochim. Acta.

[7] Costa R., Figueiredo M., Pereira C.M., Silva F. (2010). Electrochemical Double Layer at the Interfaces of Hg/Choline Chloride Based Solvents. Electrochim. Acta.

[8] Smith E.L., Abbott A.P., Ryder K.S. (2014). Deep Eutectic Solvents (DESs) and Their Applications. Chem. Rev..

[9] Cunha S.C., Fernandes J.O. (2018). Extraction Techniques with Deep Eutectic Solvents. Trends Anal. Chem..

[10] Hansen B.B., Spittle S., Chen B., Poe D., Zhang Y., Klein J.M., Horton A., Adhikari L., Zelovich T., Doherty B.W. (2021). Deep Eutectic Solvents: A Review of Fundamentals and Applications. Chem. Rev..

[11] Liu Y., Friesen J.B., McAlpine J.B., Lankin D.C., Chen S.-N., Pauli G.F. (2018). Natural Deep Eutectic Solvents: Properties, Applications, and Perspectives. J. Nat. Prod..

[12] El Achkar T., Greige-Gerges H., Fourmentin S. (2021). Basics and Properties of Deep Eutectic Solvents: A Review. Environ. Chem. Lett..

[13] Zainal-Abidin M.H., Hayyan M., Ngoh G.C., Wong W.F., Looi C.Y. (2019). Emerging Frontiers of Deep Eutectic Solvents in Drug Discovery and Drug Delivery Systems. J. Control. Release.

[14] Huang C., Chen X., Wei C., Wang H., Gao H. (2021). Deep Eutectic Solvents as Active Pharmaceutical Ingredient Delivery Systems in the Treatment of Metabolic Related Diseases. Front. Pharmacol..

[15] Liu C., Shi C., Mao F., Xu Y., Liu J., Wei B., Zhu J., Xiang M., Li J. (2014). Discovery of New Imidazole Derivatives Containing the 2,4-Dienone Motif with Broad-Spectrum Antifungal and Antibacterial Activity. Molecules.

[16] Handa M., Almalki W.H., Shukla R., Afzal O., Altamimi A.S.A., Beg S., Rahman M. (2022). Active Pharmaceutical Ingredients (APIs) in Ionic Liquids: An Effective Approach for API Physiochemical Parameter Optimization. Drug Discov. Today.

[17] Musiał M., Zorębski E., Malarz K., Kuczak M., Mrozek-Wilczkiewicz A., Jacquemin J., Dzida M. (2021). Cytotoxicity of Ionic Liquids on Normal Human Dermal Fibroblasts in the Context of Their Present and Future Applications. ACS Sustain. Chem. Eng..

[18] Shamsuri A.A. (2011). Ionic Liquids: Preparations and Limitations. Makara J. Sci..

[19] Hu L.-X., Xiong Q., Shi W.-J., Huang G.-Y., Liu Y.-S., Ying G.-G. (2021). New Insight into the Negative Impact of Imidazolium-Based Ionic Liquid [C10mim]Cl on Hela Cells: From Membrane Damage to Biochemical Alterations. Ecotoxicol. Environ. Saf..

[20] Tomé L.I.N., Baião V., da Silva W., Brett C.M.A. (2018). Deep Eutectic Solvents for the Production and Application of New Materials. Appl. Mater. Today.

[21] Paiva A., Craveiro R., Aroso I., Martins M., Reis R.L., Duarte A.R.C. (2014). Natural Deep Eutectic Solvents–Solvents for the 21st Century. ACS Sustain. Chem. Eng..

[22] Yadav A., Trivedi S., Rai R., Pandey S. (2014). Densities and Dynamic Viscosities of (Choline Chloride+glycerol) Deep Eutectic Solvent and Its Aqueous Mixtures in the Temperature Range (283.15–363.15)K. Fluid Phase Equilib..

[23] van den Bruinhorst A., Costa Gomes M. (2022). Is There Depth to Eutectic Solvents?. Curr. Opin. Green Sustain. Chem..

[24] Stefanovic R., Ludwig M., Webber G.B., Atkin R., Page A.J. (2017). Nanostructure, Hydrogen Bonding and Rheology in Choline Chloride Deep Eutectic Solvents as a Function of the Hydrogen Bond Donor. Phys. Chem. Chem. Phys..

[25] Araujo C.F., Coutinho J.A.P., Nolasco M.M., Parker S.F., Ribeiro-Claro P.J.A., Rudić S., Soares B.I.G., Vaz P.D. (2017). Inelastic Neutron Scattering Study of Reline: Shedding Light on the Hydrogen Bonding Network of Deep Eutectic Solvents. Phys. Chem. Chem. Phys..

[26] Zhang H., Lu X., González-Aguilera L., Ferrer M.L., Del Monte F., Gutiérrez M.C. (2021). Should Deep Eutectic Solvents Be Treated as a Mixture of Two Components or as a Pseudo-Component?. J. Chem. Phys..

[27] Abbott A.P., Barron J.C., Ryder K.S., Wilson D. (2007). Eutectic-Based Ionic Liquids with Metal-Containing Anions and Cations. Chem. Eur. J..

[28] Stott P. (1998). Transdermal Delivery from Eutectic Systems: Enhanced Permeation of a Model Drug, Ibuprofen. J. Control. Release.

[29] Duarte A.R.C., Ferreira A.S.D., Barreiros S., Cabrita E., Reis R.L., Paiva A. (2017). A Comparison between Pure Active Pharmaceutical Ingredients and Therapeutic Deep Eutectic Solvents: Solubility and Permeability Studies. Eur. J. Pharm. Biopharm..

[30] Aroso I.M., Silva J.C., Mano F., Ferreira A.S.D., Dionísio M., Sá-Nogueira I., Barreiros S., Reis R.L., Paiva A., Duarte A.R.C. (2016). Dissolution Enhancement of Active Pharmaceutical Ingredients by Therapeutic Deep Eutectic Systems. Eur. J. Pharm. Biopharm..

[31] Zakrewsky M., Banerjee A., Apte S., Kern T.L., Jones M.R., Sesto R.E.D., Koppisch A.T., Fox D.T., Mitragotri S. (2016). Choline and Geranate Deep Eutectic Solvent as a Broad-Spectrum Antiseptic Agent for Preventive and Therapeutic Applications. Adv. Healthc. Mater..

[32] Banerjee A., Ibsen K., Iwao Y., Zakrewsky M., Mitragotri S. (2017). Transdermal Protein Delivery Using Choline and Geranate (CAGE) Deep Eutectic Solvent. Adv. Healthc. Mater..

[33] Tanner E.E.L., Ibsen K.N., Mitragotri S. (2018). Transdermal Insulin Delivery Using Choline-Based Ionic Liquids (CAGE). J. Control. Release.

[34] Wikene K.O., Bruzell E., Tønnesen H.H. (2015). Characterization and Antimicrobial Phototoxicity of Curcumin Dissolved in Natural Deep Eutectic Solvents. Eur. J. Pharm. Sci..

[35] Li Z., Lee P.I. (2016). Investigation on Drug Solubility Enhancement Using Deep Eutectic Solvents and Their Derivatives. Int. J. Pharm..

[36] Maag H., Stella V.J., Borchardt R.T., Hageman M.J., Oliyai R., Maag H., Tilley J.W. (2007). Prodrugs of Carboxylic Acids. Prodrugs.

[37] Menniti F.S., Knoth J., Diliberto E.J. (1986). Role of Ascorbic Acid in Dopamine Beta-Hydroxylation. The Endogenous Enzyme Cofactor and Putative Electron Donor for Cofactor Regeneration. J. Biol. Chem..

[38] Leiros H.-K.S., Kozielski-Stuhrmann S., Kapp U., Terradot L., Leonard G.A., McSweeney S.M. (2004). Structural Basis of 5-Nitroimidazole Antibiotic Resistance. J. Biol. Chem..

[39] Mazaleuskaya L.L., Theken K.N., Gong L., Thorn C.F., FitzGerald G.A., Altman R.B., Klein T.E. (2015). PharmGKB Summary: Ibuprofen Pathways. Pharmacogenet. Genomics.

[40] Borges A., Ferreira C., Saavedra M.J., Simões M. (2013). Antibacterial Activity and Mode of Action of Ferulic and Gallic Acids Against Pathogenic Bacteria. Microb. Drug Resist..

[41] Aldulaimi O. (2017). General Overview of Phenolics from Plant to Laboratory, Good Antibacterials or Not. Pharmacogn. Rev..

[42] Rico-Munoz E., Bargiota E.E., Davidson P.M. (1987). Effect of Selected Phenolic Compounds on the Membrane-Bound Adenosine Triphosphatase of *Staphylococcus aureus*. Food Microbiol..

[43] Miklasińska-Majdanik M., Kępa M., Wojtyczka R., Idzik D., Wąsik T. (2018). Phenolic Compounds Diminish Antibiotic Resistance of *Staphylococcus aureus* Clinical Strains. Int. J. Environ. Res. Public Health.

[44] Rutherford S.T., Bassler B.L. (2012). Bacterial Quorum Sensing: Its Role in Virulence and Possibilities for Its Control. Cold Spring Harb. Perspect. Med..

[45] Asfour H. (2018). Anti-Quorum Sensing Natural Compounds. J. Microsc. Ultrastruct..

[46] Amrutha B., Sundar K., Shetty P.H. (2017). Effect of Organic Acids on Biofilm Formation and Quorum Signaling of Pathogens from Fresh Fruits and Vegetables. Microb. Pathog..

[47] Silva J.M., Silva E., Reis R.L., Duarte A.R.C. (2019). A Closer Look in the Antimicrobial Properties of Deep Eutectic Solvents Based on Fatty Acids. Sustain. Chem. Pharm..

[48] Silva J.M., Pereira C.V., Mano F., Silva E., Castro V.I.B., Sá-Nogueira I., Reis R.L., Paiva A., Matias A.A., Duarte A.R.C. (2019). Therapeutic Role of Deep Eutectic Solvents Based on Menthol and Saturated Fatty Acids on Wound Healing. ACS Appl. Bio Mater..

[49] Radošević K., Čanak I., Panić M., Markov K., Bubalo M.C., Frece J., Srček V.G., Redovniković I.R. (2018). Antimicrobial, Cytotoxic and Antioxidative Evaluation of Natural Deep Eutectic Solvents. Environ. Sci. Pollut. Res..

[50] Al-Akayleh F., Khalid R.M., Hawash D., Al-Kaissi E., Al-Adham I.S.I., Al-Muhtaseb N., Jaber N., Al-Remawi M., Collier P.J. (2022). Antimicrobial Potential of Natural Deep Eutectic Solvents. Lett. Appl. Microbiol..

[51] Zeng C., Liu Y., Ding Z., Xia H., Guo S. (2021). Physicochemical Properties and Antibacterial Activity of Hydrophobic Deep Eutectic Solvent-in-Water Nanoemulsion. J. Mol. Liq..

[52] Kitahara T., Koyama N., Matsuda J., Aoyama Y., Hirakata Y., Kamihira S., Kohno S., Nakashima M., Sasaki H. (2004). Antimicrobial Activity of Saturated Fatty Acids and Fatty Amines against Methicillin-Resistant *Staphylococcus aureus*. Biol. Pharm. Bull..

[53] Desbois A.P., Smith V.J. (2010). Antibacterial Free Fatty Acids: Activities, Mechanisms of Action and Biotechnological Potential. Appl. Microbiol. Biotechnol..

[54] Flemming H.-C., Wingender J. (2010). The Biofilm Matrix. Nat. Rev. Microbiol..

[55] Nava-Ocampo M.F., Fuhaid L.A., Verpoorte R., Choi Y.H., van Loosdrecht M.C.M., Vrouwenvelder J.S., Witkamp G.J., Farinha A.S.F., Bucs S.S. (2021). Natural Deep Eutectic Solvents as Biofilm Structural Breakers. Water Res..

[56] Park E.-S., Moon W.-S., Song M.-J., Kim M.-N., Chung K.-H., Yoon J.-S. (2001). Antimicrobial Activity of Phenol and Benzoic Acid Derivatives. Int. Biodeterior. Biodegrad..

[57] Zhu Y.-J., Zhou H.-T., Hu Y.-H., Tang J.-Y., Su M.-X., Guo Y.-J., Chen Q.-X., Liu B. (2011). Antityrosinase and Antimicrobial Activities of 2-Phenylethanol, 2-Phenylacetaldehyde and 2-Phenylacetic Acid. Food Chem..

[58] Wei Q., Wang X., Cheng J.-H., Zeng G., Sun D.-W. (2018). Synthesis and Antimicrobial Activities of Novel Sorbic and Benzoic Acid Amide Derivatives. Food Chem..

[59] Carrascal J.J., Pinal R., Carvajal T., Pérez L.D., Baena Y. (2021). Benzoic Acid Complexes with Eudragit E100^®^: New Alternative Antimicrobial Preservatives. Int. J. Pharm..

[60] Cook S.D. (2019). An Historical Review of Phenylacetic Acid. Plant Cell Physiol..

[61] Kim Y., Cho J.-Y., Kuk J.-H., Moon J.-H., Cho J.-I., Kim Y.-C., Park K.-H. (2004). Identification and Antimicrobial Activity of Phenylacetic Acid Produced by Bacillus Licheniformis Isolated from Fermented Soybean, Chungkook-Jang. Curr. Microbiol..

[62] Park H.J., Prausnitz M.R. (2015). Lidocaine-Ibuprofen Ionic Liquid for Dermal Anesthesia. AIChE J..

[63] Pereira C.V., Silva J.M., Rodrigues L., Reis R.L., Paiva A., Duarte A.R.C., Matias A. (2019). Unveil the Anticancer Potential of Limomene Based Therapeutic Deep Eutectic Solvents. Sci. Rep..

[64] Ahmed Arafa W.A. (2019). Deep Eutectic Solvent for an Expeditious Sono-Synthesis of Novel Series of *Bis*-Quinazolin-4-One Derivatives as Potential Anti-Cancer Agents. R. Soc. Open Sci..

[65] Lu C., Cao J., Wang N., Su E. (2016). Significantly Improving the Solubility of Non-Steroidal Anti-Inflammatory Drugs in Deep Eutectic Solvents for Potential Non-Aqueous Liquid Administration. MedChemComm.

[66] Hwang B.K., Lim S.W., Kim B.S., Lee J.Y., Moon S.S. (2001). Isolation and In Vivo and In Vitro Antifungal Activity of Phenylacetic Acid and Sodium Phenylacetate from *Streptomyces humidus*. Appl. Environ. Microbiol..

[67] Sánchez-Maldonado A.F., Schieber A., Gänzle M.G. (2011). Structure-Function Relationships of the Antibacterial Activity of Phenolic Acids and Their Metabolism by Lactic Acid Bacteria: Antibacterial Phenolic Acids. J. Appl. Microbiol..

[68] Schultheiss N., Newman A. (2009). Pharmaceutical Cocrystals and Their Physicochemical Properties. Cryst. Growth Des..

[69] Choi Y.H., van Spronsen J., Dai Y., Verberne M., Hollmann F., Arends I.W.C.E., Witkamp G.-J., Verpoorte R. (2011). Are Natural Deep Eutectic Solvents the Missing Link in Understanding Cellular Metabolism and Physiology?. Plant Physiol..

[70] Dai Y., van Spronsen J., Witkamp G.-J., Verpoorte R., Choi Y.H. (2013). Natural Deep Eutectic Solvents as New Potential Media for Green Technology. Anal. Chim. Acta.

[71] Hubbard R.E., Kamran Haider M. (2010). Hydrogen Bonds in Proteins: Role and Strength. Encyclopedia of Life Sciences.

[72] Creighton T.E. (1988). Disulphide Bonds and Protein Stability. BioEssays.

[73] Esquembre R., Sanz J.M., Wall J.G., del Monte F., Mateo C.R., Ferrer M.L. (2013). Thermal Unfolding and Refolding of Lysozyme in Deep Eutectic Solvents and Their Aqueous Dilutions. Phys. Chem. Chem. Phys..

[74] Su E., Klibanov A.M. (2015). Low-Transition-Temperature Mixtures (LTTMs) for Dissolving Proteins and for Drug Formulation. Appl. Biochem. Biotechnol..

[75] Sanchez-Fernandez A., Edler K.J., Arnold T., Alba Venero D., Jackson A.J. (2017). Protein Conformation in Pure and Hydrated Deep Eutectic Solvents. Phys. Chem. Chem. Phys..

[76] Angsantikul P., Peng K., Curreri A.M., Chua Y., Chen K.Z., Ehondor J., Mitragotri S. (2021). Ionic Liquids and Deep Eutectic Solvents for Enhanced Delivery of Antibodies in the Gastrointestinal Tract. Adv. Funct. Mater..

[77] Cruz E., Kayser V. (2019). Monoclonal Antibody Therapy of Solid Tumors: Clinical Limitations and Novel Strategies to Enhance Treatment Efficacy. BTT.

[78] Marhelava K., Pilch Z., Bajor M., Graczyk-Jarzynka A., Zagozdzon R. (2019). Targeting Negative and Positive Immune Checkpoints with Monoclonal Antibodies in Therapy of Cancer. Cancers.

[79] Adams G.P., Weiner L.M. (2005). Monoclonal Antibody Therapy of Cancer. Nat. Biotechnol..

[80] Senolt L. (2019). Emerging Therapies in Rheumatoid Arthritis: Focus on Monoclonal Antibodies. F1000Res.

[81] Isaacs J.D., Hale G., Cobbold S.P., Waldmann H., Watts R.A., Hazleman B.L., Keogan M.T. (1992). Humanised Monoclonal Antibody Therapy for Rheumatoid Arthritis. Lancet.

[82] Haraźna K., Walas K., Urbańska P., Witko T., Snoch W., Siemek A., Jachimska B., Krzan M., Napruszewska B.D., Witko M. (2019). Polyhydroxyalkanoate-Derived Hydrogen-Bond Donors for the Synthesis of New Deep Eutectic Solvents. Green Chem..

